# Quantitative morphokinetic parameters identify novel dynamics of oocyte meiotic maturation and cumulus expansion^†^

**DOI:** 10.1093/biolre/ioac139

**Published:** 2022-07-08

**Authors:** Chanakarn Suebthawinkul, Elnur Babayev, Luhan Tracy Zhou, Hoi Chang Lee, Francesca E Duncan

**Affiliations:** Department of Obstetrics and Gynecology, Feinberg School of Medicine, Northwestern University, Chicago, IL, USA; Department of Obstetrics and Gynecology, Faculty of Medicine, Chulalongkorn University, Bangkok, Thailand; Department of Obstetrics and Gynecology, Feinberg School of Medicine, Northwestern University, Chicago, IL, USA; Department of Obstetrics and Gynecology, Feinberg School of Medicine, Northwestern University, Chicago, IL, USA; Department of Obstetrics and Gynecology, Feinberg School of Medicine, Northwestern University, Chicago, IL, USA; Department of Obstetrics and Gynecology, Feinberg School of Medicine, Northwestern University, Chicago, IL, USA

**Keywords:** oocyte maturation, time-lapse, meiotic progression, cumulus expansion, embryoscope, morphokinetics, in vitro maturation

## Abstract

Meiotic maturation and cumulus expansion are essential for the generation of a developmentally competent gamete, and both processes can be recapitulated in vitro. We used a closed time-lapse incubator (EmbryoScope+™) to establish morphokinetic parameters of meiotic progression and cumulus expansion in mice and correlated these outcomes with egg ploidy. The average time to germinal vesicle breakdown (GVBD), time to first polar body extrusion (PBE), and duration of meiosis I were 0.91 ± 0.01, 8.82 ± 0.06, and 7.93 ± 0.06 h, respectively. The overall rate of cumulus layer expansion was 0.091 ± 0.002 μm/min, and the velocity of expansion peaked during the first 8 h of in vitro maturation (IVM) and then slowed. IVM of oocytes exposed to Nocodazole, a microtubule disrupting agent, and cumulus oocyte complexes (COCs) to 4-methylumbelliferone, a hyaluronan synthesis inhibitor, resulted in a dose-dependent perturbation of morphokinetics, thereby validating the system. The incidence of euploidy following IVM was >90% for both denuded oocytes and intact COCs. No differences were observed between euploid and aneuploid eggs with respect to time to GVBD (0.90 ± 0.22 vs. 0.97 ± 0.19 h), time to PBE (8.89 ± 0.98 vs. 9.10 ± 1.42 h), duration of meiosis I (8.01 ± 0.91 vs. 8.13 ± 1.38 h), and overall rate and kinetics of cumulus expansion (0.089 ± 0.02 vs 0.088 ± 0.03 μm/min) (*P* > 0.05). These morphokinetic parameters provide novel quantitative and non-invasive metrics for the evaluation of meiotic maturation and cumulus expansion and will enable screening compounds that modulate these processes.

## Teaser

We defined baseline morphokinetic parameters of mouse oocyte maturation and cumulus expansion in vitro using a closed time-lapse incubator and validated the findings using tool compounds known to disrupt these biological processes.

## Graphical Abstract

**Graphical Abstract:**
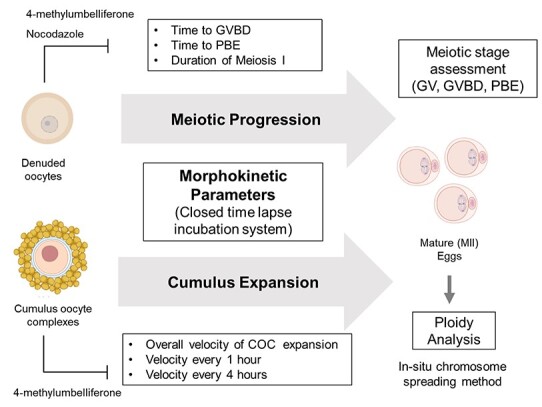

## Introduction

Ovulation, induced by the luteinizing hormone (LH) surge, involves a series of coordinated events that are necessary for the rupture and release of a mature gamete from the ovulatory follicle and into the oviduct or fallopian tube where fertilization will take place. Beyond the rupture of the follicle wall, two major events of ovulation include the final stages of oocyte maturation and expansion of the cumulus layer that surround the oocyte [1, 2]. In response to the LH surge, signaling pathways induce the oocyte to resume meiosis and transition from the diakinesis stage of prophase of meiosis I (MI) to metaphase of meiosis II (MII), which is characterized by nuclear (germinal vesicle) envelope breakdown (GVBD), meiotic spindle assembly, rearrangement of the cortical cytoskeleton, and extrusion of the first polar body (PBI) [2–5]. Concomitant with the changes in the oocyte, the surrounding cumulus cells also undergo maturation-associated changes. The LH surge induces the cumulus layer to expand through increased production and accumulation of hyaluronan (HA) and other extracellular matrix components [6–10]. The volumetric change that occurs during cumulus expansion facilitates follicle rupture and typically correlates with the progression of oocyte maturation [11, 12]. Therefore, meiotic progression and cumulus expansion are essential for oocyte developmental competence, successful ovulation, and fertilization [8, 11, 13].

Notably both the process of meiotic maturation and cumulus expansion can be fully recapitulated outside the ovary when either denuded oocytes or cumulus oocyte complexes (COCs) are matured in vitro, and hallmarks of these processes can be visualized morphologically by transmitted light microscopy [5, 14]. First, meiotic maturation is associated with the loss of the germinal vesicle (GV), or delineation of the nucleus due to germinal vesicle breakdown (GVBD), which signifies meiotic resumption and the transition of the oocyte from the prophase I arrest. The extrusion of the PBI indicates completion of MI and transition of the oocyte to the arrest at MII [1, 2]. These characteristic cellular features of meiotic maturation are morphologic readouts of the precise timing of cell cycle progression, and disturbances in meiotic maturation can result in aneuploidy and negatively impact oocyte quality [15, 16]. Concurrently, the cumulus layer undergoes a transformation from a compact structure into a dispersed one formed by the synthesis and accumulation of a prominent extracellular matrix [9, 17]. The timing of oocyte meiotic maturation, reduction of cumulus cell–oocyte coupling, and cumulus expansion are correlated, but the reduction in cumulus–oocyte coupling is not itself the cause of meiotic resumption. Nevertheless, cumulus expansion likely contributes to uncoupling between these cell types, especially in vivo [18]. Moreover, the presence of cumulus cells improves the developmental competence of the resulting gamete following in vitro and in vivo maturation [19, 20]. Defective cumulus expansion has a detrimental effect on oocyte competence such as impaired meiotic maturation and poor embryo development [11, 12].

Morphokinetics refers to time-specific morphological changes, and in the context of the final stages of oocyte maturation during ovulation, there are clear morphokinetic parameters for meiotic progression and cumulus layer expansion, which can provide dynamic information regarding the developing gamete [1]. These morphokinetics can be assessed using time-lapse imaging technology, which overcomes the logistical challenges of static image-based analysis by enabling continuous and non-invasive imaging [21–25]. Morphokinetics analysis has been widely adopted in the setting of clinical Assisted Reproductive Technology (ART) laboratories to monitor human preimplantation embryo development and to develop algorithms for embryo selection [26, 27]. For these purposes, morphokinetics are tracked in closed time-lapse monitoring systems in which temperature, humidity, and oxygen tension are tightly controlled. These systems typically consist of stand-alone incubators containing inverted microscopes coupled to digital cameras. The digital images are collected every few minutes at multiple different focal planes with low-intensity light to avoid damage to the embryos, and these images can be subsequently processed into time-lapse videos [28–30]. This technology can accommodate simultaneous and continuous monitoring of hundreds of embryos in parallel and eliminates the need to image outside of the incubator. This capability minimizes sample handling and the alteration of the culture environment, which is advantageous over the existing methods [21, 31, 32]. Although morphokinetics analysis using closed time-lapse monitoring systems provides powerful quantitative metrics regarding biological processes, it has so far been limited to preimplantation embryos in the field of reproductive science and medicine.

The goal of this study was to leverage the capacity and capabilities of the EmbryoScope+™ (Vitrolife, Denver, CO) platform to establish the morphokinetic parameters of mouse oocyte in vitro maturation (IVM), including meiotic progression and cumulus expansion. We then correlated the established parameters with the ploidy status of the resulting MII eggs. Moreover, we used tool compounds with known inhibitory effects on meiotic progression and cumulus expansion to validate the responsiveness of these parameters to perturbations. These morphokinetic parameters provide novel quantitative and non-invasive metrics for the evaluation of meiotic progression and cumulus expansion and, importantly, can be assessed in a high throughput capacity in hundreds of samples in parallel. This methodology is significant because of its diverse applications, including the study of fundamental biology, compound testing for ferto-toxicity or contraceptive drug screening, and non-invasive assessment of gamete quality in the setting of human IVM.

## Materials and methods

### Animals

Reproductively young CD1 female mice at 6–12 weeks of age were obtained from Envigo (Indianapolis, IN). Based on a linear extrapolation of age, the 6–12-week-old mice are equivalent to women in their 20s [33, 34]. Mice were housed in a controlled barrier facility at Northwestern University’s Center for Comparative Medicine in Chicago under constant temperature, humidity, and light (14 h light/10 h dark). Upon arrival at Northwestern University, mice were fed with a diet formulated to exclude soybean meal, Teklad Global 2916 chow (Envigo, Madison, WI), and were provided food and water ad libitum. All animal experiments described were approved by the Institutional Animal Care and Use Committee (Northwestern University) and performed in accordance with National Institutes of Health Guidelines.

### Ovarian hyperstimulation and COCs collection

To maximize the yield of COCs collected, mice were given intraperitoneal injections of 5 IU pregnant mare serum gonadotropin (PMSG) (ProSpec-Tany TechnoGene, East Brunswick, NJ, Cat # HOR-272), and 44–46 h post-PMSG injection, ovaries were harvested. Isolated ovaries were placed into dishes containing pre-warmed Leibovitz’s medium (L15) (Life Technologies Corporation, Grand Island, NY) supplemented with 3 mg/ml polyvinylpyrrolidone (PVP) (Sigma-Aldrich, St. Louis, MO) and 0.5% (v/v) Penicillin–Streptomycin (PS) (Life Technologies Corporation, Grand Island, NY) (L15/PVP/PS)*.* Antral follicles were mechanically punctured with insulin syringes to release COCs from the ovaries. COCs were then transferred to L15/PVP/PS medium containing 2.5 μM milrinone (Sigma-Aldrich, St. Louis, MO), a phosphodiesterase 3A (PDE3A) inhibitor that maintains oocytes arrested in prophase of MI [5]. To obtain isolated oocytes, the surrounding cumulus cells were removed from the COCs by mechanical disruption. The resulting denuded oocytes were allowed to recover in alpha-MEM + GlutaMAX (Thermo Fisher Scientific, Waltham, MA)/PS/Bovine Serum Albumin (BSA) (Sigma-Aldrich, St. Louis, MO) (alpha-MEM/PS/BSA) supplemented with 2.5 μM milrinone for 1 h at 37°C in a humidified atmosphere of 5% CO_2_ in air prior to being loaded into an EmbryoSlide (Vitrolife, Denver, CO). At least three independent replicates were performed for each experiment. The COCs or denuded oocytes were pooled together from two to four animals per experiment to minimize any animal-specific variability.

### IVM within the EmbryoSlide and EmbryoScope+™

EmbryoSlides (Vitrolife, Denver, CO) were prepared the day before the experiment to allow media in the dishes to equilibrate in the EmbryoScope+™. The 16 microwells in the EmbryoSlides, each with a diameter of ~250 μm, were filled according to the manufacturer’s instructions with the specific maturation media designated for oocytes or COCs as described below. The microwells were overlaid with 1.6 ml of mineral oil (Sigma-Aldrich, St. Louis, MO) and equilibrated in the EmbryoScope+™ for 9–24 h (Supplementary Figure S1).

Oocyte maturation was induced by the removal of milrinone, which results in the degradation of Cyclic adenosine monophosphate (cAMP) and meiotic resumption of the oocyte [1, 35]. Depending on the experiment, denuded oocytes or COCs were loaded into the wells of the EmbryoSlide according to the manufacturer’s instructions. Denuded oocytes were matured in alpha-MEM/PS/BSA media, whereas the intact COCs were matured in specific media that induces and supports cumulus expansion (alpha-MEM/5%(v/v) Fetal bovine serum (FBS)/0.02%(v/v) Epidermal growth factor (EGF)/20 mM N-(2-Hydroxyethyl)piperazine-N′-(2-ethanesulfonic acid) (HEPES)/0.25 mM pyruvate) [36, 37]. EGF, HEPES, and pyruvate were purchased from Sigma-Aldrich (St. Louis, MO) and FBS was purchased from Thermo Fisher Scientific (Waltham, MA). EmbryoSlides were then loaded into the EmbryoScope+™ (Supplementary Figure S1). Denuded oocytes or COCs were in vitro matured for a total of 16 h at 37°C in a humidified atmosphere of 5% CO_2_ in the air. Images were taken every 10 min at 11 focal planes with low-intensity red LED illumination with <0.5 s of light exposure per image. These conditions are identical to those used for humans in ART and therefore are considered to have minimal impact (if any) on gametes and preimplantation embryos. This technology can accommodate simultaneous and continuous monitoring of 240 samples and eliminates the need to image outside of the incubator.

Following IVM, the meiotic maturation status of each oocyte was assessed based on morphological criteria. For in vitro matured COCs, the surrounding cumulus cells were removed following a brief incubation in 0.25 mg/ml hyaluronidase (Sigma-Aldrich, St. Louis, MO) so that the meiotic stage of the oocyte could be accurately visualized. Oocytes that failed to mature and remained arrested at prophase of MI were characterized by an intact nucleus or germinal vesicle (GV oocyte), whereas mature eggs arrested at metaphase of meiosis II (MII egg) were characterized by extrusion of the PBI. Oocytes that had undergone GVBD but lacked a PBI were in between prophase I and MII and were referred to as a GVBD/MI oocyte. The percentage of oocytes at each stage of meiosis was reported in all experiments.

### Analysis of time-lapse data for denuded oocytes

**Figure 1:**
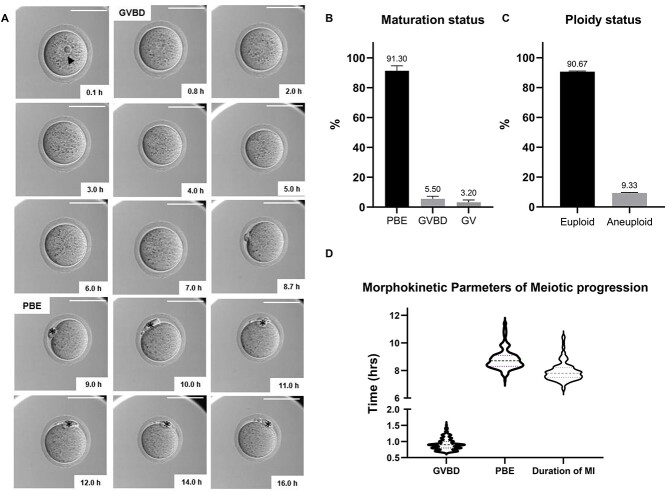
Baseline parameters of denuded oocytes during IVM. (A) A representative series of montage images show meiotic progression in an individual oocyte within EmbryoScope+™. The time when denuded oocytes were put into the EmbryoScope+™ was set as the starting point. Time to GVBD referred to the first time that we did not observe the germinal vesicle membrane (0.8 h in this oocyte), and time to PBE represented the first time when PBI membrane completely separated from the oocyte membrane (9.0 h in this oocyte). Time difference between GVBD and PBE is the duration of meiosis I, which is 8.2 h in this oocyte (scale bar =100 μm). (B) Maturation status of denuded oocytes after IVM. (C) Ploidy status of resulting MII eggs. (D) Morphokinetic parameters of meiotic progression including time to GVBD, time to PBE, and duration of meiosis I in denuded oocytes (*n* = 172, three replicates). GV, germinal vesicle; GVBD, germinal vesicle breakdown; PBE, polar body extrusion; MI, meiosis I; PBI, first polar body; IVM, in vitro maturation; arrowhead, GV; asterisk, PBI.

To establish baseline morphokinetic parameters of meiotic progression, denuded oocytes (*n* = 172, three replicates) were matured in the EmbryoScope+™. The time-lapse imaging data were evaluated using analysis software provided by the manufacturer (EmbryoViewer, Vitrolife, Denver, MO), which includes an annotation function to capture information and is intended for displaying, storing, and transferring images generated by the EmbryoScope+™. We determined the morphokinetic parameters of meiotic progression, including time to GVBD, time to first polar body extrusion (PBE), and duration of MI (Supplementary Video S1). The time when the denuded oocytes were put into the EmbryoScope+™ and imaging was started was set as *t* = 0. The time to GVBD was defined as the first time when the loss of the GV was observed, and the time to PBE was defined when cytokinesis was complete between the PBI and oocyte plasma membranes rather than when the PBI first began extrusion (Figure 1A). The duration of MI was defined as the time difference between GVBD and PBE and is the critical time frame during which the oocyte assembles a bipolar spindle, aligns its chromosomes on the metaphase I plate, and establishes proper kinetochore–microtubule attachments [22]. The time to GVBD, time to PBE, and duration of MI was determined and plotted for each individual oocyte. In addition to the morphokinetics, we also assessed the other morphological parameters of denuded oocytes manually on EmbryoViewer. For each time point, 11 images were taken through the *z*-axis. These images were carefully reviewed and the focal plane where the structure of interest was best in focus was used for analysis. The annotation function in the EmbryoViewer software was used to demarcate the structure and the area measurement for this region of interest was recorded (Supplementary Figure S2A). These parameters included nucleolar number, GV/nucleus area, oocyte area, perivitelline space (PVS) area, zona pellucida (ZP) area, and cytoplasm area of individual oocytes, which were assessed at the beginning of IVM. The cytoplasm area was calculated by subtracting the GV area from the oocyte area. PBI area was assessed at the end of IVM.

### Analysis of time-lapse data for intact COCs

To establish baseline morphokinetic parameters of cumulus expansion, COCs (*n* = 168, three replicates) were matured in the EmbryoScope+™. After IVM of COCs, morphokinetic parameters of cumulus layer expansion were evaluated with the EmbryoViewer software. Time point when COCs were put into the EmbryoScope+™ was set as the starting point. The distances of cumulus layer expansion were measured every 1 h at the same position as much as possible until the end of 16 h observation or until the cumulus layer expanded beyond the well limits (Figure 3A, Supplementary Video S1). The overall rate of cumulus layer expansion, the velocity of cumulus expansion at every 1 h, and the velocity of cumulus expansion at every 4 h were calculated by using these formulas:

$$\begin{align*} &\mathrm{Overall}\ \mathrm{rate}\ \mathrm{of}\ \mathrm{expansion}\ \left(\mu \mathrm{m}/\min \right)\\ &=\frac{\mathrm{Distance}\ \mathrm{at}\ \mathrm{the}\ \mathrm{end}\ \mathrm{of}\ \mathrm{expansion}-\mathrm{Distance}\ \mathrm{at}\ \mathrm{start}}{\mathrm{Time}\ \mathrm{at}\ \mathrm{the}\ \mathrm{end}\ \mathrm{of}\ \mathrm{expansion}-\mathrm{Time}\ \mathrm{at}\ \mathrm{start}\ } \end{align*}$$

$$\begin{align*} &\mathrm{Velocity}\ \mathrm{of}\ \mathrm{expansion}\ \mathrm{at}\ \mathrm{each}\ \mathrm{time}\ \mathrm{point}\ \left(\mu \mathrm{m}/\min \right)\\ &=\frac{\mathrm{Distance}\ \mathrm{at}\ 2\mathrm{nd}\ \mathrm{time}\ \mathrm{point}-\mathrm{Distance}\ \mathrm{at}\ 1\mathrm{st}\ \mathrm{time}\ \mathrm{point}}{\mathrm{Time}\ \mathrm{at}\ 2\mathrm{nd}\ \mathrm{point}-\mathrm{Time}\ \mathrm{at}\ 1\mathrm{st}\ \mathrm{point}}\end{align*}$$

### Validation of meiotic progression parameters with Nocodazole

Denuded oocytes (*n* = 190 oocytes, three replicates) were in vitro matured in the EmbryoScope+™ in media containing increasing doses of Nocodazole (control, 12.5, 25, 50, and 75 nM) prepared from a stock solution of 0.1 mM Nocodazole in dimethyl sulfoxide (DMSO) (Sigma-Aldrich, St. Louis, MO, Cat # M1404). The same volume of DMSO as in the 75 nM group was added to the media as the control group. These oocytes were then evaluated for maturation status and morphokinetic parameters of meiotic progression as described above.

To confirm that the Nocodazole treatment disrupted the microtubule cytoskeleton, after IVM, the oocytes were fixed in 3.8% paraformaldehyde (PFA) (Electron Microscopy Sciences, Hatfield, PA) with 0.1% TritonX-100 (TX-100) (Sigma-Aldrich, St. Louis, MO) for 20 min at 37°C. After fixation, oocytes were washed with the blocking buffer (1X PBS, 0.01% Tween-20 (Sigma-Aldrich, St. Louis, MO), 0.02% Sodium azide (NaN_3_) (Sigma-Aldrich, St. Louis, MO)_,_ and 0.3% BSA) twice for 5 min. The oocytes were then incubated in permeabilization solution (1X PBS, 0.1% TX-100, 0.02% NaN_3_, and 0.3% BSA) for 15 min at room temperature. The oocytes were washed again with blocking buffer and then incubated in alpha-tubulin (11H10) Rabbit mAB 488 (1:100; Cell Signaling Technology, Danvers, MA, Cat # 5063S) and Rhodamine Phalloidin (1:50; Invitrogen, Waltham, MA, Cat # R415) for 1 h at room temperature to visualize microtubules and actin. Stained oocytes were rinsed with blocking buffer three times for 20 min. The oocytes were then mounted on slides in Vectashield Antifade Mounting Medium with DAPI (4′,6-diamidino-2-phenylindole; Vector Laboratories, Burlingame, CA) to visualize chromosome alignment on meiotic spindles. Cells were imaged on a Leica SP5 inverted laser scanning confocal microscope (Leica Microsystems) using 405, 488, and 543 nm lasers. For meiotic spindle evaluation, the imaging was performed under 40× magnification and Z-stack thickness was 1 μm. Spindles were considered normal if they exhibited a bipolar structure with all chromosomes aligned on the metaphase plate. All images were processed using LAS AF (Leica Microsystems) and analyzed using FIJI (National Institutes of Health, Bethesda, MD).

### Validation of cumulus expansion parameters with 4-methylumbelliferone (4MU)

COCs (*n* = 172 oocytes, three replicates) were in vitro matured in EmbryoScope+™ in media with different concentrations of 4MU (Sigma-Aldrich, St. Louis, MO, Cat # M1508). The 4MU was first dissolved in water (stock solution of 30 mg/ml), and then diluted to different final concentrations (control, 0.1, 0.5, and 1 mM) in culture media. The same volume of water as in the 1 mM group was added to the media as the control group. These COCs were then evaluated for morphokinetic parameters of cumulus expansion as described above. To determine whether 4MU had direct effects on the oocyte, denuded oocytes (*n* = 172 oocytes, two replicates) were in vitro matured in the EmbryoScope+™ with the same concentrations of 4MU used for intact COCs (control, 0.1, 0.5, and 1 mM). Meiotic progression and spindle morphology were then assessed as described above.

To confirm that 4MU disrupted the HA content of COCs, an established hyaluronan-binding protein (HABP) assay was adapted for use in whole-mount staining [38–42]. In vitro matured COCs were fixed in 3.8% PFA for 30 min at room temperature. After fixation, COCs were washed in the blocking buffer twice for 5 min. Fixed COCs were then adhered to poly-D-lysine-coated glass slides (100 μg/ml of poly-D lysine in ddH_2_O) by pipetting COCs onto the slide and aspirating fluid from the periphery until COCs made contact with the slide. Endogenous avidin and biotin were blocked using an Avidin/Biotin blocking kit (Vector Laboratories, Burlingame, CA). Avidin was applied for 15 min, slides were rinsed in 1X PBS, and then biotin was applied for 15 min. Slides were rinsed in 1X PBS and were then incubated in normal goat serum (Vector Laboratories, Burlingame, CA) for 20 min. Following a wash in 1X PBS, biotinylated HABP (Calbiochem, San Diego, CA, Cat # 38599) diluted in normal goat serum (1:100) was added to all sections for 1 h incubation at room temperature. Slides were then washed in 1X PBS. Signal was amplified by incubating slides in ABC reagent (Vector Laboratories, Burlingame, CA) for 30 min followed by TSA Plus Fluorescein System (Akoya Biosciences, Marlborough, MA). Then samples were mounted in Vectashield Antifade Mounting Medium with DAPI to stain cell nuclei. COCs were imaged on a Leica SP5 inverted laser scanning confocal microscope using 405 and 488 nm lasers. For the HA analysis, the imaging was performed under 40× magnification and Z-stack thickness was 1.5 μm. HA content was measured as the mean intensity of signal per COC area. All images were processed using LAS AF and analyzed using FIJI.

### Ploidy analysis

Following IVM, resulting MII eggs were evaluated for ploidy status using an in situ chromosome spreading method [43]. Importantly, all oocytes were stained and tracked individually throughout the experiment so that the ploidy data could be directly correlated with the morphokinetic and morphological parameters. MII eggs were first treated with 100 μM monastrol (Tocris Bioscience, Bristol, UK), which collapses the bipolar spindle into a monopolar one and results in the dispersion of the chromosomes within an intact cell [44]. This incubation was performed at 37°C in a humidified atmosphere of 5% CO_2_ in the air for 3 h. The eggs were fixed in 2% PFA for 20 min at room temperature. After fixation, the eggs were washed with the blocking buffer twice for 5 min. Then they were treated with the permeabilization solution for 15 min at room temperature and were washed again with the blocking buffer. To detect kinetochores, the eggs were incubated with the primary antibody (1:200 Human anti-Centromere/Kinetochore, Antibodies Incorporated, Davis, CA, Cat # 15-234) at 4°C overnight. The cells were then rinsed with the blocking buffer three times for 20 min and incubated with the secondary antibody (1:100, goat anti-human IgG (H + L) AlexaFluor 488, Invitrogen, Waltham, MA, Cat # A-11013) for 1 h at room temperature. Then the eggs were washed again with the blocking buffer three times and mounted in Vectashield Antifade Mounting Medium with DAPI. Eggs were imaged on a Leica SP5 inverted laser scanning confocal microscope using 405 and 488 nm lasers. For the kinetochore analysis, the imaging was performed under 100× magnification and Z-stack thickness was 0.5 μm [45]. Ploidy status was evaluated by manually counting the kinetochores in each *z*-plane through a stack encompassing the entire oocyte. Two investigators blinded to the experimental conditions performed the counting. A euploid mouse egg contains a total of 20 pairs of sister chromatids with 40 kinetochores, and any egg that differed from these numbers was considered aneuploid. All images were processed using LAS AF and analyzed using FIJI.

### Statistical analysis

Data are presented as the mean ± standard error of the mean, and each experiment was repeated three times. All results were graphed using Graphpad Prism Software Version 8.0.1 (La Jolla, CA). The normal distribution of data was evaluated with the Shapiro–Wilk test. Analysis between groups of continuous variables was performed with Student’s *t*-test or Mann–Whitney *U* test. Multiple comparisons were analyzed with one-way analysis of variance (ANOVA) test, Kruskal–Wallis test, and two-way ANOVA (mixed-effects analysis) followed by Tukey’s multiple comparison tests. Categorical variables were analyzed with Fisher’s exact test or Chi-square test. The correlation between continuous variables was analyzed with the Pearson Correlation test. *P* values <0.05 were considered statistically significant. Bonferroni correction was used to adjust the *P* value when multiple comparisons were made using Fisher’s exact or Chi-square tests.

## Results

### Baseline morphokinetic and morphologic parameters of denuded oocytes during IVM

To determine baseline morphokinetic parameters of meiotic progression during spontaneous oocyte maturation, we matured denuded oocytes collected from reproductively young mice in the EmbryoScope+™ (Figure 1A, Supplementary Video S1). We observed that 91.30 ± 3.35% of denuded oocytes underwent PBE within the EmbryoScope+™, whereas 3.20 ± 1.60% of cells remained arrested in prophase I (GV) and 5.50 ± 1.78% of cells were either in pro-metaphase I or metaphase I (GVBD) (Figure 1B). Under these conditions, the incidence of euploid and aneuploid eggs was 90.67 ± 0.41 and 9.33 ± 0.41%, respectively (Figure 1C). For oocytes that progressed to PBE, we evaluated time to GVBD, time to PBE, and duration of MI, which were 0.91 ± 0.19, 8.82 ± 0.75, and 7.93 ± 0.70 h, respectively (Table 1, Figure 1D).

**Table 1:** Baseline parameters of denuded oocytes and COCs during IVM in closed time-lapse incubator, and the comparison of these parameters between euploid and aneuploid eggs (mean ± SEM)

**Denuded oocytes**				
**Parameters**	**Overall (*n* = 172)**	**Euploid (*n* = 137)**	**Aneuploid (*n* = 13)**	***P* value**
PBE rate (%)	91.30 ± 3.35			
Euploidy rate (%)	90.67 ± 0.41			
Morphological parameters				
GV area (μm^2^)	428.05 ± 2.48	432.18 ± 3.00	419.91 ± 5.42	0.212
Oocyte area (μm^2^)	4236.21 ± 28.15	4283.82 ± 32.36	4134.0 ± 76.35	0.169
PVS area (μm^2^)	762.28 ± 31.33	754.21 ± 34.60	899.18 ± 139.5	0.317
ZP area (μm^2^)	2015.66 ± 21.96	2051.48 ± 23.33	2073.36 ± 91.41	0.787
PBI area (μm^2^)	429.95 ± 6.26	430.23 ± 7.31	425.91 ± 31.64	0.555
Cytoplasm area (μm^2^)	3808.16 ± 27.47	3851.64 ± 31.64	3751.50 ± 78.83	0.328
Morphokinetic parameters				
Time to GVBD (h)	0.91 ± 0.01	0.90 ± 0.01	0.97 ± 0.06	0.168
Time to PBE (h)	8.82 ± 0.06	8.89 ± 0.08	9.10 ± 0.11	0.739
Duration of meiosis I (h)	7.93 ± 0.06	8.01 ± 0.07	8.13 ± 0.11	0.764
**Cumulus–oocyte complexes**				
**Parameters**	**Overall (*n* = 168)**	**Euploid (*n* = 139)**	**Aneuploid (*n* = 10)**	***P* value**
PBE rate (%)	96.16 ± 2.34			
Euploidy rate (%)	93.54 ± 0.93			
Overall rate of expansion (μm/min)	0.091 ± 0.002	0.091 ± 0.002	0.089 ± 0.007	0.737

IVM, in vitro maturation; GV, germinal vesicle; PVS, perivitelline space; ZP, zona pellucida; PBI, first polar body; GVBD, germinal vesicle breakdown; PBE, polar body extrusion; COCs, cumulus–oocyte complexes; SEM, standard error of the mean.

In addition to the morphokinetic parameters, we also assessed morphologic parameters (Supplementary Figure S2). The average area of the GV, the oocyte, the PVS, the ZP, the PBI, and cytoplasm were 428.05 ± 2.48, 4236.21 ± 28.15, 762.28 ± 31.33, 2015.66 ± 21.96, 429.95 ± 6.26, and 3808.16 ± 27.47 μm^2^, respectively (Table 1, Supplementary Figure S2A and B). We examined whether there were any correlations between these morphological parameters and observed a strong correlation between the cytoplasm and oocyte areas (*r* = 0.996, *P* < 0.0001). There were also significant correlations between the cytoplasm and ZP areas (*r* = 0.393, *P* < 0.0001), and the oocyte and ZP areas (*r* = 0.395, *P* < 0.0001) (Supplementary Figure S2C–F). We also evaluated whether there were associations between these morphological parameters and morphokinetic parameters of meiotic progression, but did not observe any strong correlation (Supplementary Figure S3).

The nucleolus is the organelle responsible for ribosome biogenesis, and the mammalian oocyte has a prominent nucleolus given the high level of translation in the oocyte during its growth phase [46, 47] (Figure 1A, Supplementary Figure S4). Given that nucleoli are clearly visible within the oocyte nucleus by transmitted light microscopy, we evaluated whether there was a relationship between nucleolar number and morphokinetic parameters of meiotic progression. In all, 80.23% of denuded oocytes had one nucleolus, 11.04% had two nucleoli, and the remaining 8.73% had between three and nine nucleoli (Supplementary Figure S4A and B). There were no differences in the ability of the oocytes to reach MII stage (89.78 vs. 94.12%, *P* = 0.742) or in the incidence of euploidy in the resulting eggs (90.48 vs. 91.67%, *P* > 0.99) based on nucleolar number (1 nucleolus and >1 nucleoli) (Supplementary Figure S4C and D). There were also no differences in the time to GVBD (0.93 ± 0.19 vs. 0.87 ± 0.14 h), time to PBE (8.83 ± 0.75 vs. 8.62 ± 0.51 h), and duration of MI (7.95 ± 0.72 vs. 7.75 ± 0.45 h) between denuded oocytes with 1 nucleolus and >1 nucleoli (*P* > 0.05) (Supplementary Table S1, Supplementary Figure S4E–G). We further evaluated the morphological parameters between these two groups of oocytes. Although there were no significant differences in the GV area (428.0 ± 2.75 vs. 428.7 ± 5.92 μm^2^), oocyte area (4233.19 ± 33.35 vs. 4261.18 ± 45.88 μm^2^), ZP area (2013.76 ± 24.80 vs. 2038.74 ± 46.51 μm^2^), and cytoplasm area (3805.17 ± 32.46 vs. 3832.44 ± 45.88 μm^2^), the area of the PVS (736.75 ± 21.64 vs. 495.40 ± 60.86 μm^2^, *P* < 0.0001) and PBI (436.78 ± 6.93 vs. 402.61 ± 13.63 μm^2^, *P* = 0.0085) were significantly larger in oocytes with 1 nucleolus than in oocytes with >1 nucleoli (Supplementary Table S1, Supplementary Figure S4H–M).

### Nocodazole treatment disrupts the microtubule cytoskeleton and morphokinetic parameters of meiotic maturation

**Figure 2:**
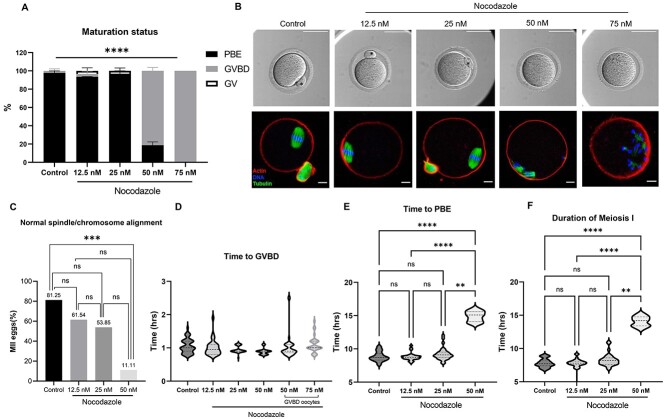
Nocodazole inhibits oocyte maturation, spindle formation, and meiotic progression in a dose-dependent manner. (A) Nocodazole significantly impairs the maturation of denuded oocytes in a dose-dependent manner. (B, upper panel) A representative of individual oocyte after incubation with different concentrations of Nocodazole. The majority of the oocytes exposed to 50 and 75 nM of Nocodazole were arrested at the GVBD stage (asterisk, PBI) (scale bars = 100 μm). (B, lower panel) A representative *z*-projections of meiotic spindle (tubulin, green) and chromosome (DNA, blue) from oocytes exposed to different concentrations of Nocodazole (actin, red). Images show normal spindle formation and chromosome alignment in oocytes exposed to lower concentrations (control, 12.5, 25, and 50 nM) and abnormal spindle formation in oocytes exposed to higher concentrations (50 and 75 nM) of Nocodazole (scale bars = 10 μm). (C) Nocodazole significantly disrupts normal spindle formation and normal chromosome alignment in a dose-dependent manner. (D–F) Morphokinetic parameters of meiotic progression in oocytes exposed to different concentrations of Nocodazole (*n* = 190, three replicates). (ns; *P* > 0.05, ^*^^*^P < 0.01, ^*^^*^^*^P < 0.001, ^*^^*^^*^^*^P < 0.0001); GV, germinal vesicle; GVBD, germinal vesicle breakdown; PBE, polar body extrusion; MI, meiosis I; MII, metaphase of meiosis II.

To validate the established morphokinetic parameters of meiotic progression, we in vitro matured denuded oocytes in Nocodazole, a specific microtubule disruptor that perturbs cell division [48–53]. We deliberately used Nocodazole as a tool compound with well-documented effects on oocyte meiotic progression and spindle formation to demonstrate that our morphokinetic parameters were responsive to perturbations. Nocodazole impaired the maturation of denuded oocytes in a dose-dependent manner. Although the proportion of oocytes that reached the MII stage were similar among the control, 12.5, and 25 nM groups (97.78 ± 2.22, 93.54 ± 0.21, 96.88 ± 3.13%, respectively, *P* > 0.05), these percentages were significantly lower in the 50 and 75 nM groups (18.75 ± 3.61 and 0%, respectively) compared with controls (*P* < 0.0001). The majority of the oocytes exposed to 50 and 75 nM of Nocodazole had undergone GVBD but not extruded a PBI and thus were considered in MI (Figure 2A). Although meiotic progression was similar in the control, 12.5, and 25 nM Nocodazole groups, the resulting eggs differed with respect to their microtubule cytoskeleton (Figure 2B and C). Whereas 81.25% of the eggs in the control group had normal bipolar MII spindles with chromosomes aligned on the metaphase plate, this was reduced to 61.54 and 53.85% in the 12.5 and 25 nM Nocodazole groups, respectively (*P* = 0.0012) (Figure 2C). The severity of this phenotype was much more pronounced and significantly impaired in the 50 nM group where only 11.11% of the eggs had a normal spindle (Figure 2A–C, Supplementary Figure S5). The lack of mature eggs with the 75 nM Nocodazole exposure precluded our ability to analyze MII spindles in this group.

To determine whether the microtubule perturbations induced by Nocodazole treatment were associated with altered morphokinetic parameters of meiotic progression, we analyzed the timing of GVBD, time to PBE, and duration of MI across experimental cohorts (Figure 2D–F). Nocodazole treatment did not affect the time of GVBD (1.05 ± 0.03, 1.01 ± 0.04, 0.95 ± 0.02, 1.02 ± 0.06, and 1.10 ± 0.04 h for the control, 12.5, 25, 50, and 75 nM Nocodazole, respectively, *P* = 0.252) (Figure 2D, Supplementary Table S2, Supplementary Video S2). In contrast, time to PBE was delayed by ~5 h in the 50 nM Nocodazole group relative to control and lower doses of the drug (14.89 ± 0.26 h for 50 nM Nocodazole vs. 8.85 ± 0.11, 8.81 ± 0.11, 9.22 ± 0.17 h for control, 12.5, 25 nM Nocodazole, respectively, *P* < 0.0001; Figure 2E and Supplementary Table S2, Supplementary Video S2). The extended time to PBE in response to 50 nM Nocodazole treatment translated to a longer duration of MI in this cohort relative to the others (14.08 ± 0.25 h vs. 7.81 ± 0.09, 7.60 ± 0.28, 8.27 ± 0.16 h for control, 12.5, and 25 nM Nocodazole, respectively, *P* < 0.0001; Figure 2F, Supplementary Table S2). These results demonstrate the dose-dependent inhibitory effect of Nocodazole on oocyte maturation and meiotic spindle formation. Importantly, this analysis allowed us to identify conditions in which PBIs were extruded but which were still associated with significant spindle and chromosome abnormalities (50 nM Nocodazole). In these instances, such cellular defects were paralleled by significantly altered morphokinetics, which could be tracked non-invasively. Therefore, these findings validate the use of morphokinetic parameters as a non-invasive and sensitive readout for the detection of perturbations that compromise egg quality.

### Baseline morphokinetic parameters of cumulus expansion in COCs during IVM

**Figure 3:**
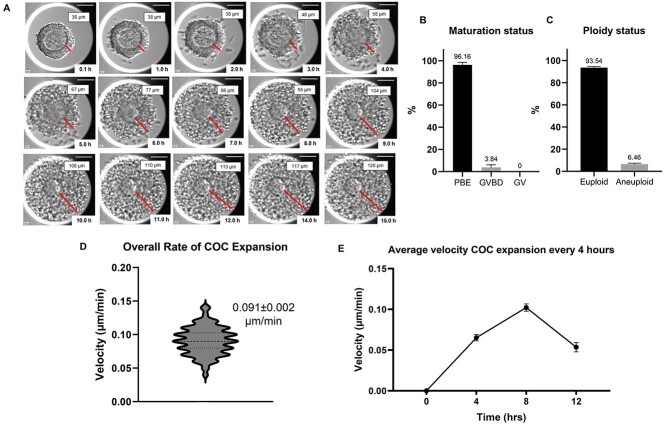
Baseline parameters of COCs during IVM. (A) A representative series of montage images show cumulus layer expansion of an individual COC within EmbryoScope+™. The time when COCs were put into the EmbryoScope+™ was set as the starting point. The distances of cumulus layer expansion were measured every 1 h at the same position as much as possible until the end of the 16 h observation or until the cumulus layer expanded beyond the well limits (scale bar =100 μm). (B) Maturation status of COCs after IVM. (C) Ploidy status of the resulting MII eggs. (D and E) Morphokinetic parameters of cumulus expansion including (D) overall rate of cumulus expansion and (E) velocity of expansion every 4 h (*n* = 168, three replicates). COCs, cumulus–oocyte complexes; IVM, in vitro maturation; GV, germinal vesicle; GVBD, germinal vesicle breakdown; PBE, polar body extrusion.

To evaluate the baseline morphokinetic parameters of cumulus expansion, we matured intact COCs in the EmbryoScope+™ (Figure 3A, Supplementary Video S1). Following IVM in this system, 96.16 ± 4.0% of oocytes within intact COCs reached the MII stage, and 93.54 ± 0.93% of these cells were euploid, demonstrating optimal culture conditions (Figure 3B and C). There were no significant differences in maturation rate and ploidy status between denuded oocytes and COCs (*P* > 0.05) (Figure 1B and C). We then assessed the overall rate of cumulus layer expansion, the average velocity of cumulus layer expansion at every 1 h, and the average velocity of cumulus layer expansion at every 4 h (Figure 3D and E, Supplementary Figure S6A). The overall rate of cumulus layer expansion was 0.091 ± 0.021 μm/min (Table 1, Figure 3D). When the velocity of cumulus layer expansion was analyzed at every 1 h or every 4 h across the entire maturation period, we observed a highly reproducible dynamics of expansion whereby cumulus expansion rate increased during the first 8 h of IVM and then subsequently decreased (Figure 3E, Supplementary Figure S6A, Supplementary Video S1). Interestingly, the timing of this change in cumulus layer expansion rate correlates with extrusion of the PBI (Figures 1D and 3E, Supplementary Figure S6A).

### 4MU inhibits HA production, cumulus expansion, and oocyte maturation in a dose-dependent manner

**Figure 4:**
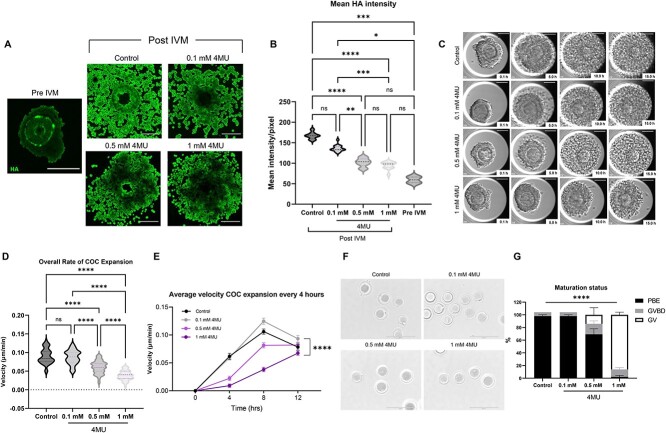
4MU inhibits HA production, cumulus expansion, and oocyte maturation in a dose-dependent manner. (A) Representative *z*-projections of HA level analysis of individual COC in pre-IVM and after incubation with different concentrations of 4MU (HA, green) (scale bars = 100 μm). (B) Mean intensity of HA signal per pixel in pre-IVM COC and in COCs incubated with different concentrations of 4MU. (C) Representative individual COC during incubation with different concentrations of 4MU (scale bars = 100 μm). (D and E) Morphokinetic parameters of cumulus expansion in COCs incubated with different concentrations of 4MU including (D) overall rate of cumulus expansion and (E) velocity of expansion every 4 h. (F) Representative individual oocyte (after denudation) after incubation with different concentrations of 4MU. The majority of the oocytes exposed to 0.5 and 1 mM of 4MU were arrested at the GV stage (scale bars = 400 μm). (G) 4MU significantly impairs the oocyte maturation in a dose-dependent manner (*n* = 172, three replicates). (ns; *P* > 0.05, ^*^P < 0.05, ^*^^*^P < 0.01, ^*^^*^^*^P < 0.001, ^*^^*^^*^^*^P < 0.0001); 4MU, 4-Methylumbelliferone; COCs, cumulus–oocyte complexes; HA, hyaluronan; IVM, in vitro maturation; GV, germinal vesicle; GVBD, germinal vesicle breakdown; PBE, polar body extrusion.

To validate the established morphokinetic parameters of cumulus expansion, we used an inhibitor of HA synthesis, 4MU, which is required for cumulus expansion [54–56]. 4MU acts as a competitive substrate for Uridine 5′-diphospho-(UDP)-glucuronosyl transferase, an enzyme involved in HA synthesis, and reduces the expression of *Has* mRNA [57]. To validate the established morphokinetic parameters of cumulus expansion, we first determined whether 4MU could efficiently inhibit the production of the HA matrix in the COC. As expected, the baseline level of HA in COCs was low pre-IVM but increased significantly post-IVM concurrent with cumulus expansion (Figure 4A and B). HA is synthesized on the cytosolic side of the cell membrane by membrane-embedded hyaluronan synthases and then released into the extracellular space [58]. During meiotic maturation, there is an increase in hyaluronan synthase 2 (*Has2*) expression in cumulus cells, which mediates production of HA, thereby explaining the intensity of the HABP signal at the cell surface of cumulus cells [59, 60]. 4MU treatment decreased HA levels in COCs in a dose-dependent manner (Figure 4A and B). The mean intensity of fluorescent HA signal per pixel of COC image was 167.1 ± 2.88, 136.0 ± 1.85, 101.8 ± 2.76, and 94.68 ± 2.50 in the control, 0.1, 0.5, 1 mM 4MU groups, respectively (*P* < 0.0001, Figure 4A and B, Supplementary Table S2). Importantly, COCs post-IVM that had been treated with 0.5 and 1 mM 4MU had similar levels of HA as pre-IVM COCs (Figure 4A and B). Of note, the reduction of HA is most prominent closest to the oocyte, and this could be due to an imaging artifact if cumulus expansion was impaired with 4MU treatment, resulting in thicker samples relative to controls (Figure 4A). To exclude this possibility, we evaluated the thickness of COCs that were imaged and did not observe a difference across the experimental cohorts (19.69 ± 1.28,19.50 ± 1.05, 21.45 ± 1.50, and 19.65 ± 1.04 μm for control, 0.1, 0.5, and 1 mM 4MU groups, respectively, *P* = 0.683). Therefore, the reduction of HA that occurs in a dose-dependent manner with increasing doses of 4MU is unlikely to be an artifact. A biological explanation for why HA is still observed at the periphery of COCs even following 4MU treatment is that inhibition is partial. Therefore, some expansion still occurs, and this is coincident with the presence of HA.

To determine whether morphokinetic parameters of cumulus expansion were responsive to perturbation of the HA matrix, we in vitro matured COCs in increasing doses of 4MU (Figure 4C). The overall rates of cumulus layer expansion decreased in a dose-dependent manner (0.090 ± 0.003, 0.087 ± 0.003, 0.060 ± 0.003, and 0.038 ± 0.002 μm/min in control, 0.1, 0.5, and 1 mM 4MU groups, respectively, *P* < 0.0001; Figure 4D, Supplementary Table S2, Supplementary Video S3). Similar to the overall expansion, the average velocity of cumulus expansion every 1 and 4 h across the maturation period differed among the groups in a dose-dependent manner when comparing the 0.1, 0.5, and 1 mM 4MU treatment groups (*P* < 0.0001, Figure 4E, Supplementary Figure S6B, Supplementary Table S2).

To further understand the impact of 4MU on oocyte maturation, we assessed the meiotic stage of oocytes following IVM (Figure 4F and G). The oocytes appeared morphologically normal by transmitted light microscopy, demonstrating that there was no overt cellular toxicity of 4MU (Figure 4F). Interestingly, however, meiotic progression was inhibited in a dose-dependent manner. The percentage of cells that underwent PBE was 97.92 ± 2.08, 97.92 ± 2.08, 69.30 ± 8.67, and 2.22 ± 2.22% in control, 0.1, 0.5, and 1 mM 4MU groups, respectively (*P* < 0.0001, Figure 4G, Supplementary Table S2). The maturation rate was significantly decreased in COCs exposed to 0.5 and 1 mM of 4MU relative to controls, and the majority of the oocytes exposed to 1 mM 4MU remained arrested at the GV stage (*P* < 0.0001, Figure 4G, Supplementary Table S2). These results demonstrate a dose-dependent inhibitory effect of 4MU on HA synthesis, which is reflected in altered morphokinetic parameters of cumulus expansion. Thus, our data suggest an association between inhibition of cumulus expansion and compromised meiotic progression, which is consistent with what has been observed in the bovine model where HA-mediated COC expansion was inhibited with 4MU and in the porcine where it was inhibited by 6-diazo-5-oxo-l-norleucine (DON) [54, 61].

**Figure 5:**
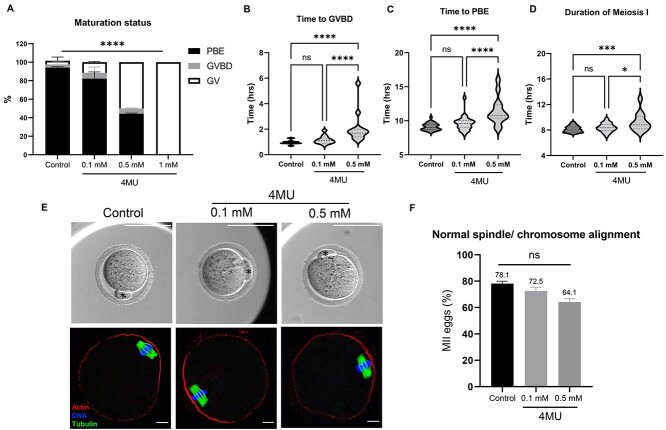
4MU perturbs oocyte maturation and morphokinetic parameters of meiotic progression in denuded oocytes. (A) 4MU significantly impairs the denuded oocyte maturation. (B–D) Morphokinetic parameters of meiotic progression in denuded oocytes exposed to different concentrations of 4MU, including (B) time to GVBD, (C) time to PBE, and (D) duration of MI. (E, upper panel) A representative of individual oocyte after incubation with different concentrations of 4MU (asterisk, PBI) (scale bars = 100 μm). (E, lower panel) A representative *z*-projections of meiotic spindle (tubulin, green) and chromosome (DNA, blue) from oocytes exposed to different concentrations of 4MU (actin, red) (scale bars = 10 μm). (F) Normal spindle formation and normal chromosome alignment are similar in resulting MII eggs incubated with different concentrations of 4MU (*n* = 172, two replicates). (ns; *P* > 0.05, ^*^P < 0.05, ^*^^*^^*^P < 0.001, ^*^^*^^*^^*^P < 0.0001); 4MU, 4-Methylumbelliferone; GV, germinal vesicle; GVBD, germinal vesicle breakdown; PBE, polar body extrusion; MI, meiosis I; MII, metaphase of meiosis II.

To determine whether the effect of 4MU on meiotic maturation could be due to direct effects on the oocyte, we in vitro matured denuded oocytes in the presence of 4MU. We observed a dose-dependent inhibition in meiotic maturation. The percentage of oocytes that underwent PBE was 94.17 ± 0.84, 82.24 ± 7.24, 44.38 ± 0.63, and 0% in control, 0.1, 0.5, and 1 mM 4MU groups, respectively, and the majority of the oocytes exposed to higher concentrations of 4MU remained arrested at the GV stage (*P* < 0.0001, Figure 5A). This pattern is similar to that observed when intact COCs were in vitro matured (Figure 4G). To determine whether 4MU treatment was associated with altered morphokinetic parameters of meiotic progression, we analyzed the time to GVBD, time to PBE, and duration of MI across experimental groups (Figure 5B–D). These morphokinetic parameters of meiotic progression were all significantly prolonged in oocytes treated with 50 mM 4MU compared with lower doses of the drug and the control (Supplementary Table S2, Figure 5B–D, *P* < 0.0001). In the cells that did extrude a polar body following IVM, there was no difference between those that had been treated with 4MU compared with controls in terms of normal spindle morphology (Supplementary Table S2, Figure 5E and F, *P* = 0.365). However, there was a trend towards decreased normal spindle morphology and chromosome alignment with increasing concentrations of 4MU (78.10 ± 1.91, 72.50 ± 2.50, and 64.10 ± 2.56% for control, 0.1, and 0.5 mM 4MU treated oocytes, respectively). Thus, 4MU has direct effects on the oocyte in addition to the cumulus cells.

### Morphokinetic parameters of meiotic progression and cumulus expansion are similar between euploid and aneuploid eggs from reproductively young mice

**Figure 6:**
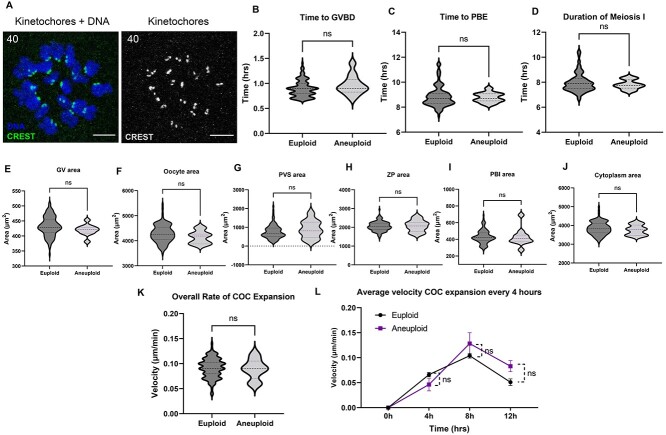
Morphokinetic and morphological parameters are similar between euploid and aneuploid eggs from reproductively young mice. (A) Representative maximum *z*-projections of in situ chromosome spreads from a euploid egg (kinetochores (CREST), green or gray scale) (DNA, blue) (scale bars = 5 μm). (B–D) Morphokinetic parameters of meiotic progression of euploid and aneuploid eggs. (E–J) Morphological parameters of euploid and aneuploid eggs (euploid *n* = 137 eggs, aneuploid =13 eggs, three replicates). (K–M) Morphokinetic parameters of cumulus expansion of euploid and aneuploid eggs (euploid *n* = 139 eggs, aneuploid =10 eggs, three replicates): (K) overall rate of cumulus expansion and (L) the velocity of expansion every 4 h (ns; *P* > 0.05); GV, germinal vesicle; PVS, perivitelline space; ZP, zona pellucida; PBI, first polar body; GVBD, germinal vesicle breakdown; PBE, polar body extrusion; MI, meiosis I; MII, metaphase of meiosis II; COCs, cumulus–oocyte complexes; IVM, in vitro maturation.

Although we expected Nocodazole and 4MU to have quantitative effects on meiotic progression and cumulus expansion, respectively, especially at higher doses, we wanted to determine whether the morphologic and morphokinetics parameters we established were responsive to more subtle physiologic differences in egg quality. Thus, following IVM of either denuded oocytes or intact COCs, we assessed ploidy status in the resulting MII eggs using an in situ chromosome spreading method (Figure 6A) and then stratified our analysis of morphokinetic parameters of meiotic progression and cumulus expansion based on euploid and aneuploid eggs (Figure 6). When IVM was performed with denuded oocytes, the incidence of euploid and aneuploid eggs were 90.67 ± 0.41 (*n* = 137) and 9.33 ± 0.41% (*n* = 13), respectively (Figure 1C, Table 1). There were no differences in the morphokinetic parameters of meiotic maturation between euploid and aneuploid eggs, including time to GVBD (0.90 ± 0.22 vs. 0.97 ± 0.19 h), time to PBE (8.89 ± 0.98 vs. 9.10 ± 1.42 h), and duration of MI (8.01 ± 0.91 vs. 8.13 ± 1.38 h) (*P* > 0.05, Figure 6A–D, Table 1). Similar to the morphokinetics, morphological parameters were also not different between euploid and aneuploid eggs: GV area (432.18 ± 3.00 vs. 419.91 ± 5.42 μm^2^), oocyte area (4283.82 ± 32.36 vs. 4134.0 ± 76.35 μm^2^), PVS area (754.21 ± 34.60 vs. 899.18 ± 139.5 μm^2^), ZP area (2051.48 ± 23.33 vs. 2073.36 ± 91.41 μm^2^), PBI area (430.23 ± 7.31 vs. 425.91 ± 31.64 μm^2^), and cytoplasm area (3851.64 ± 31.64 vs. 3751.50 ± 78.83 μm^2^) (*P* > 0.05, Figure 6E–J, Table 1).

When IVM was performed with intact COCs, the incidence of euploidy was 93.54 ± 0.93% (*n* = 139) and aneuploid eggs was 6.46 ± 0.93% (*n* = 10) (Figure 3C, Table 1). There was no difference in the overall rate of cumulus layer expansion between COCs that ultimately produced euploid and aneuploid eggs (0.089 ± 0.02 vs. 0.088 ± 0.03 μm/min, respectively, *P* > 0.05, Table 1, Figure 6K). The average velocity of cumulus expansion every 1 h or every 4 h across the maturation period were also similar between the euploid and aneuploid cohorts (*P* > 0.05, Figure 6L, Supplementary Figure S6C). The kinetics of expansion was similar between euploid and aneuploid eggs with faster expansion during the first 8 h, which then slowed. Overall, there were no significant differences in the parameters we examined between euploid and aneuploid eggs; however, there is a limitation of a low incidence of aneuploidy in the reproductively young population.

## Discussion

To the best of our knowledge, this is the first morphokinetic analysis of mouse IVM of both denuded oocytes and intact COCs using a state-of-the-art time-lapse technology (EmbryoScope+™), which is typically used in human ART [28, 32]. Although live imaging of oocyte maturation is not novel and has been successfully performed by numerous research groups, the system used here provides a highly stable and safe culture environment of temperature, gas concentration, and humidity that is approved for use with human embryos [23, 62]. Moreover, this system has the potential to be high throughput, given that hundreds of samples can be monitored continuously and simultaneously without the need for removal from the incubator for up to days. The system, however, is only capable of transmitted light microscopy, and thereby is limited to non-invasive assessment of cellular morphology and morphokinetic parameters. In addition, the field of view is limited to 200 μm, and therefore, studies cannot currently be performed with larger structures such as ovarian follicles during in vitro follicle growth [63–65].

In our study, we used oocytes from reproductively young mice, which based on a linear extrapolation are equivalent to women in their 20s to establish the baseline morphological and morphokinetic parameters of meiotic progression and cumulus expansion during IVM [34]. The range of maturation rate in our study was 91–96%, which appears higher relative to previously published studies of IVM performed in conventional incubators which report maturation rates ranging from 75 to 90% in mice of comparable ages [22, 50]. IVM of intact COCs showed a trend towards higher meiotic maturation rates compared with denuded oocytes, and this observation is consistent with the well-established role of cumulus cells in supporting this process [11, 66–68]. Morphokinetic parameters of meiotic progression including time to GVBD, time to PBE, and duration of MI were ~0.75–1.5 h shorter compared with what has previously been reported [5, 22, 50, 53]. For example, in oocytes from B6D2F1/J mice that were matured in vitro, the average time to GVBD, time to PBE, and duration of MI were 1.92 ± 0.06, 10.48 ± 0.11, and 8.68 ± 0.10 h, respectively [22]. In MF1 mice, oocytes required 1–2 and 10–12 h to complete GVBD and MI, respectively [53]. The differences in the timing of these meiotic progression events between studies could be due to different IVM systems used (standard vs. biphasic IVM) [69] and the inherent biological differences between mouse strains [22, 50, 53, 70]. However, these findings could also be because closed time-lapse incubator systems provide a more stable culture environment with consistent maintenance of the optimal temperature and gas concentrations and may phenocopy in vivo processes better than traditional incubators [30, 71].

In reproductively young mice, the incidence of aneuploidy in studies using conventional incubators ranged from 8 to 18% [22, 52, 72], and those following maturation in vivo were 3–10% [73, 74]. Thus, the incidence of aneuploidy in the EmbryoScope+™ of 6–9% appears to be more consistent with what occurs in vivo. Together, the high maturation and low aneuploidy rates observed in our study suggest that the closed time-lapse incubator technology (EmbryoScope+™) may be a more optimal environment for IVM. However, differences in animal strain and age as well as variation in culture conditions (e.g., media, incubators, oxygen tension) could significantly influence results. Therefore, future studies comparing in vivo maturation to IVM in both conventional and closed time-lapse incubators in parallel are needed to draw firm conclusions.

A striking and unanticipated observation of our imaging analysis was the dynamic behavior of cumulus cells, with the kinetics of cumulus expansion being faster and peaking during the first 8 h of IVM compared with later time periods. This is consistent with previous studies demonstrating that genes involved in this process, including *Has2*, prostaglandin endoperoxide synthase 1, 2 (*Ptgs1, Ptgs2*), and tumor necrosis factor-alpha-induced protein 6 (*Tnfaip6*) are highly expressed at 4–8 h post-IVM or ovulation induction, and their levels then gradually decrease [59, 60]. Our finding also corroborates work showing that the ability of cumulus cells to migrate is low in unexpanded COCs but increases steadily until reaching a peak at ovulation [75]. Furthermore, we observed that the timing of this change in cumulus layer expansion rate correlates with the timing of PBI extrusion. Thus, it is tempting to speculate that there is tight coordination between the oocyte and the surrounding somatic cells at this stage, with the oocyte potentially releasing a signal at the time of PBI extrusion that causes a change in behavior of cumulus cells. In fact, cumulus expansion at the time of ovulation is regulated by both the gonadotropin surge and oocyte-secreted factors such as growth and differentiation factor 9, bone morphogenetic protein 15, EGF, and cumulus expansion enabling factor [4, 11, 76–78]. Whether oocytes of specific meiotic stages have differential paracrine effects on cumulus cell gene expression and subsequent behavior is an important research area.

In addition to uncovering new biology, analysis of morphokinetics during oocyte maturation can serve as an important platform for screening compounds that perturb meiotic progression and cumulus expansion. Therefore, we validated our established morphokinetic parameters using specific tool compounds. Given its known mechanism-of-action as a microtubule disruptor, we expected to observe meiotic arrest at metaphase I or a prolonged duration of MI as well as increased spindle and chromosomes segregation abnormalities in oocytes treated with Nocodazole, and in fact, we observed dose-dependent effects on these parameters [22, 49, 50]. The timing to GVBD was not affected, since spindle formation occurs after GVBD, which is the target of microtubule inhibition via Nocodazole [22, 50]. The most variable phenotypes were observed in oocytes treated with 50 nM Nocodazole. Although 18.75 ± 3.61% of oocytes exposed to 50 nM Nocodazole underwent PBI extrusion, only 11.11% of the resulting eggs had morphologically normal bipolar MII spindles and chromosome alignment on the metaphase plate. Importantly, meiotic progression was significantly prolonged, as evidenced by increased time to PBE and duration of MI. Thus, major defects in meiotic progression can be detected non-invasively via significant changes in morphokinetic parameters.

The inhibition of cumulus expansion with 4MU in our system also demonstrated a dose-dependent response, and this was paralleled by a decrease in the percentage of oocytes that progressed through meiosis and reached the mature MII-arrested stage. This inhibition of meiotic maturation could be due to indirect effects on the oocyte via the cumulus cells, direct effects on the oocyte, or a combination of both. Interestingly, 4MU inhibited meiotic maturation in a dose-dependent manner with nearly all oocytes remaining arrested at the GV-intact stage when treated with 1 mM 4MU and almost half the oocytes when treated with 0.5 mM 4MU. Although these oocytes did not progress through meiosis, they were morphologically normal and there was no evidence of overt toxicity as occurs, for example, when oocytes are exposed to plastic leachates [79]. We recently demonstrated that oocytes express hyaluronan synthase 3 (*Has3*), and 4MU has been shown to inhibit HA synthesis by depletion of cellular UDP-glucuronic acid [39, 80]. Thus, it is possible that 4MU is inhibiting oocyte-specific HA production. 4MU treatment also prolonged all morphokinetic parameters of meiotic progression in denuded oocytes in a dose-responsive manner. Although spindle formation and chromosome alignment were normal in the resulting MII eggs across experimental cohorts, there was a tendency towards abnormal configurations in the higher concentrations of 4MU. Further studies are needed to determine the function of oocyte-derived HA, and especially the potential role of intracellular HA on meiotic maturation [81]. Interestingly, studies in mouse, rabbit, and hamster demonstrate that denuded oocytes can produce and secrete HA, which regulates the enlargement of the PVS and influences polyspermy at fertilization [55, 56].

Overall, in our study, the association of spindle and chromosome segregation defects with prolonged morphokinetic parameters of meiotic progression was observed with both Nocodazole and 4MU, underscoring the potential of morphokinetic parameters as non-invasive metrics to differentiate abnormal oocytes. These data demonstrate that this time-lapse system provides quantitative morphokinetic parameters of meiotic progression, which are sensitive to perturbations and that go beyond the simple binary assessment of meiotic maturation and cumulus expansion. Moreover, the EmbryoScope+™ system can accommodate simultaneous monitoring of 240 COCs or oocytes with up to 15 conditions. Therefore, it can be applied to screen compounds to evaluate effects of endocrine disruptors, ferto- and gonado-toxic chemicals, and potential non-hormonal contraceptives.

In addition to a screening tool, the system and morphologic parameters established here may have clinical relevance for human IVM performed in the setting of fertility preservation [82–84]. In pre-pubertal girls or post-pubertal females who do not have time for ovarian stimulation due to clinical urgency, ovarian tissue cryopreservation is a potential fertility preservation option. In this method, the ovarian cortex containing primordial follicles is preserved, and during tissue processing, small antral follicles are disrupted releasing COCs, which can be collected and used for IVM [83]. This method has been used successfully to obtain mature gametes from females of various ages, and numerous live births have also been reported [83–85]. As far as we are aware, human IVM and its associated morphokinetic parameters have not been established in the EmbryoScope+™ system, but this represents a potentially unique opportunity to develop new methods to select the highest quality gametes from the standpoints of meiotic and cytoplasmic competence, especially if coupled to automated analyses. Importantly, the analysis of biological data from closed time-lapse live imaging systems are improving, and these platforms are increasingly becoming integrated with artificial intelligence technologies to analyze the vast amount of morphological and morphokinetic results and/or evaluate the acquired visual material via computer vision to select the gametes and embryos with the highest developmental potential [86].

Although most chromosome segregation errors occur during MI [6, 73, 87, 88], we did not observe differences in morphological and morphokinetic parameters of meiotic progression between euploid and aneuploid eggs, which is consistent with previously published studies that also did not observe any difference in time to GVBD, time to PBE, and duration of MI in individual oocytes based on ploidy status [22, 53]. Furthermore, our results are similar to morphokinetic and morphological studies in the human embryo, which also did not find significant differences in time-lapse parameters and morphological appearances between euploid and aneuploid blastocysts [89–91]. Similar to the meiotic progression, we found no differences in morphokinetic parameters of cumulus layer expansion between euploid and aneuploid eggs. However, the number of aneuploid eggs analyzed in this study was small due to the low incidence of chromosome segregation errors in reproductively young mice, so it is possible that we were not able to identify significant changes due to insufficient statistical power. Studies are ongoing with mice of advanced reproductive age where the incidence of aneuploidy is higher. However, the developmental potential of the egg is dictated by both nuclear and cytoplasmic competence [92, 93]. Thus, it is also possible that the morphokinetic parameters are predominantly influenced by the cytoplasmic competence of the oocyte. Future work will focus on evaluating morphokinetic parameters of meiotic maturation and cumulus expansion and how they correlate with the implantation and developmental potential of the resulting embryos.

In summary, the use of a state-of-the-art closed time-lapse incubator with the integrated imaging system (EmbryoScope+™) allowed us to establish and validate morphokinetic parameters of mouse oocyte meiotic progression and cumulus expansion. We were able to track the oocytes individually, and rigorously correlate the morphokinetic features with the ploidy outcomes. Although there were no differences in morphokinetic parameters of oocyte maturation in regards to the ploidy status, the established parameters provide new quantitative insights into meiotic progression and cumulus expansion. Monitoring oocyte maturation morphokinetics in a stable, non-invasive, and high-throughput closed time-lapse imaging system has significant implications for understanding the fundamental biology of oocyte maturation and cumulus expansion, use as a screening tool to assess compounds for ferto-toxicity or contraceptive potential, and for gamete selection in clinical human IVM.

## Data availability

All original data in this publication are available upon request to the corresponding author.

## Authors’ contributions

FED conceived the original idea. CS designed and carried out the experiments. CS collected, analyzed, and interpreted data and wrote the original manuscript. CS, EB, and FED provided critical discussion, reviewed, and revised manuscript. EB, HL, LTZ, and FED helped supervise the project. All authors provided a final approval for the manuscript before publication.

## Supplementary Material

Suebthawinkul-supplemantal_figures-1_ioac139

Suebthawinkul-supplemantal_figures-2_ioac139

Suebthawinkul-supplemantal_figures-3_ioac139

Suebthawinkul-supplemantal_figures-4_ioac139

Suebthawinkul-supplemantal_figures-5_ioac139

Suebthawinkul-supplemantal_figures-6_ioac139

Suebthawinkul_Supplemental_Table_ioac139

Supplemental_Video_1_ioac139

Supplemental_Video_2_ioac139

Supplemental_Video_3_ioac139
