# Supplementary figures and images for: Quantitative morphokinetic parameters identify novel dynamics of oocyte meiotic maturation and cumulus expansion
Source: Biol Reprod. 2022 Jul 8;107(4):1097–112. doi: 10.1093/biolre/ioac139 (PMC9562117; doi:10.1093/biolre/ioac139)

# Supplemental Figure 1

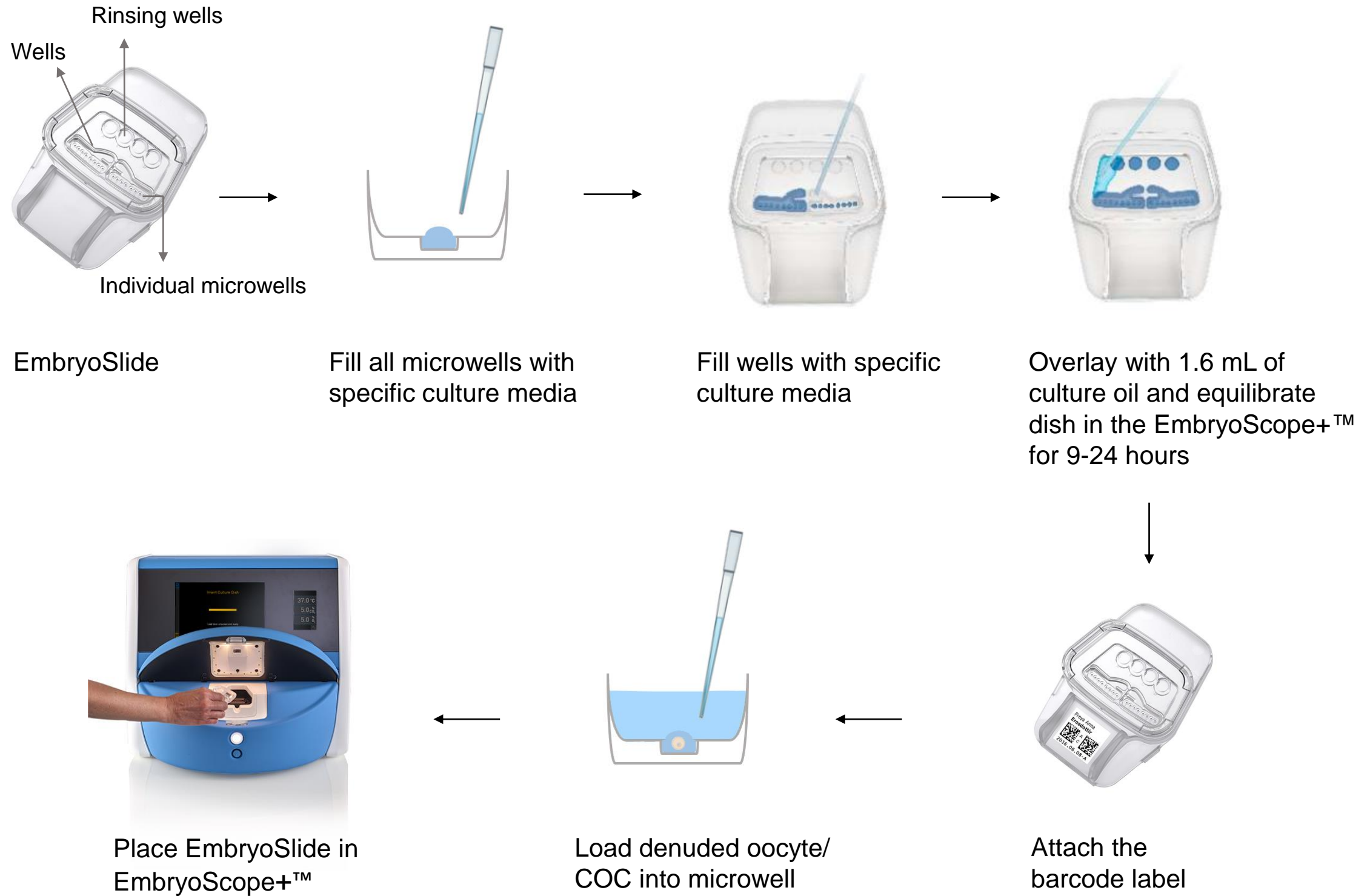

Supplement: Suebthawinkul-supplemantal_figures-1_ioac139 [file suebthawinkul-supplemantal_figures-1_ioac139.pdf]

Supplemental Figure 2

A

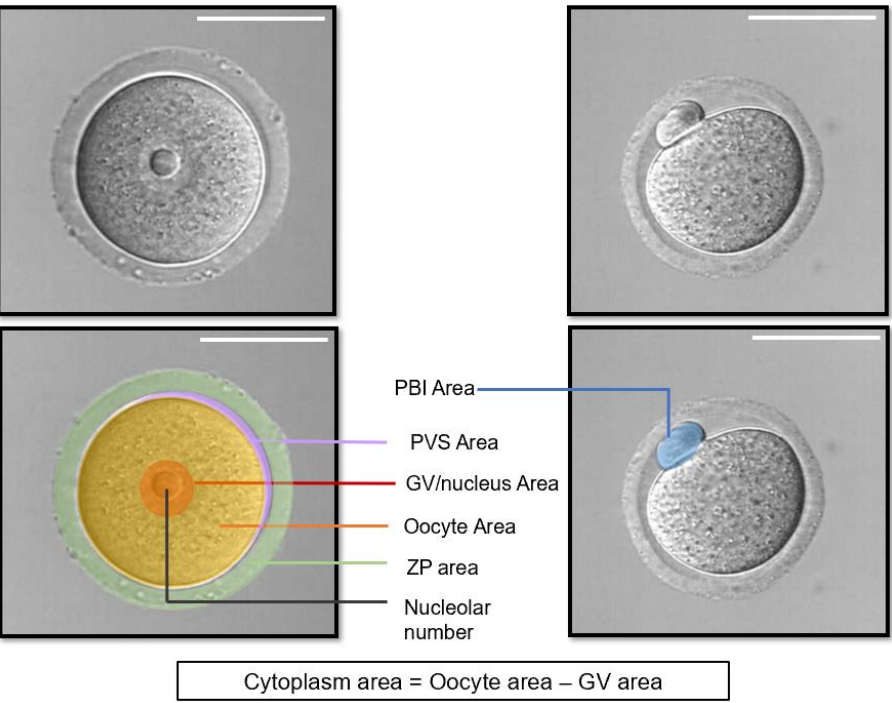

B

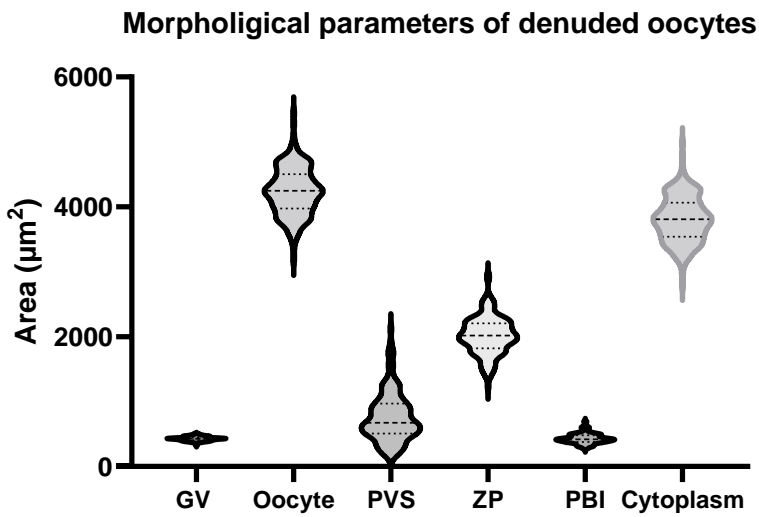

C

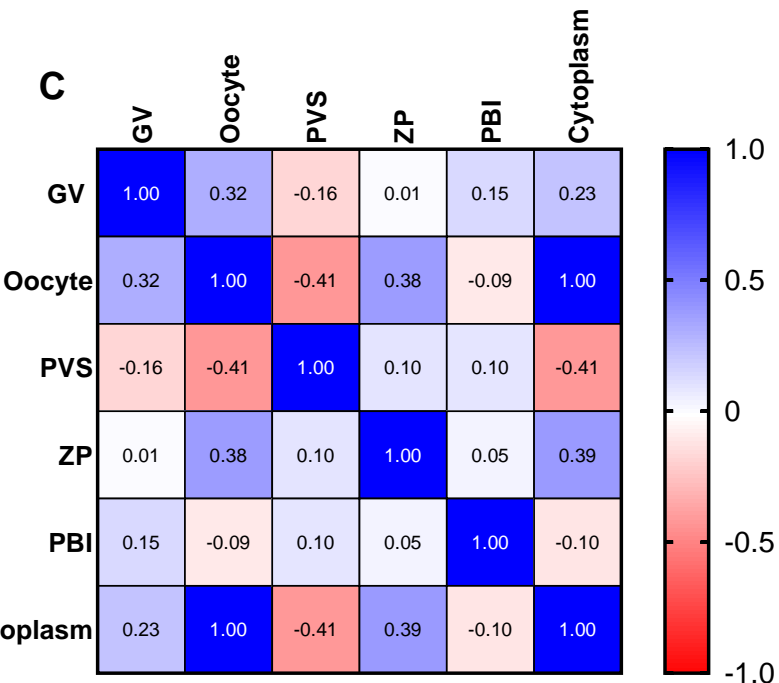

D

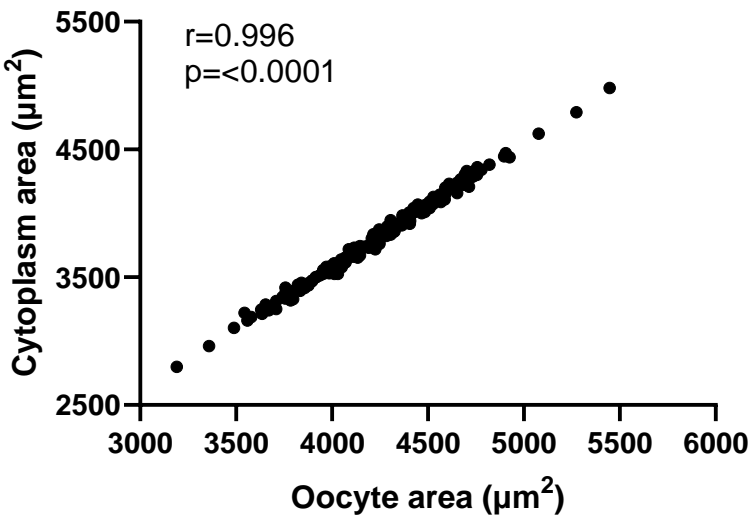

E

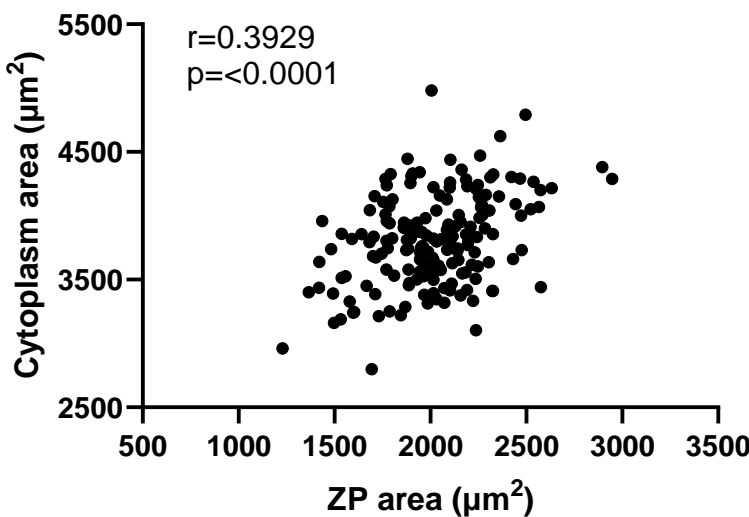

F

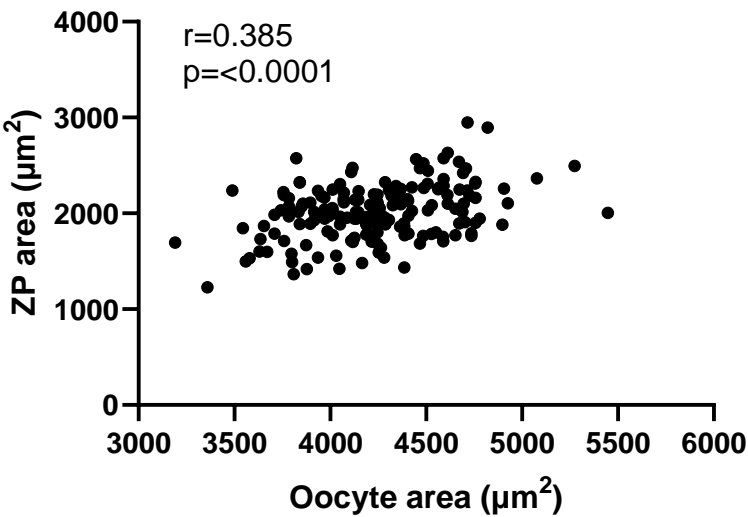

Supplement: Suebthawinkul-supplemantal_figures-2_ioac139 [file suebthawinkul-supplemantal_figures-2_ioac139.pdf]

Supplemental Figure 3

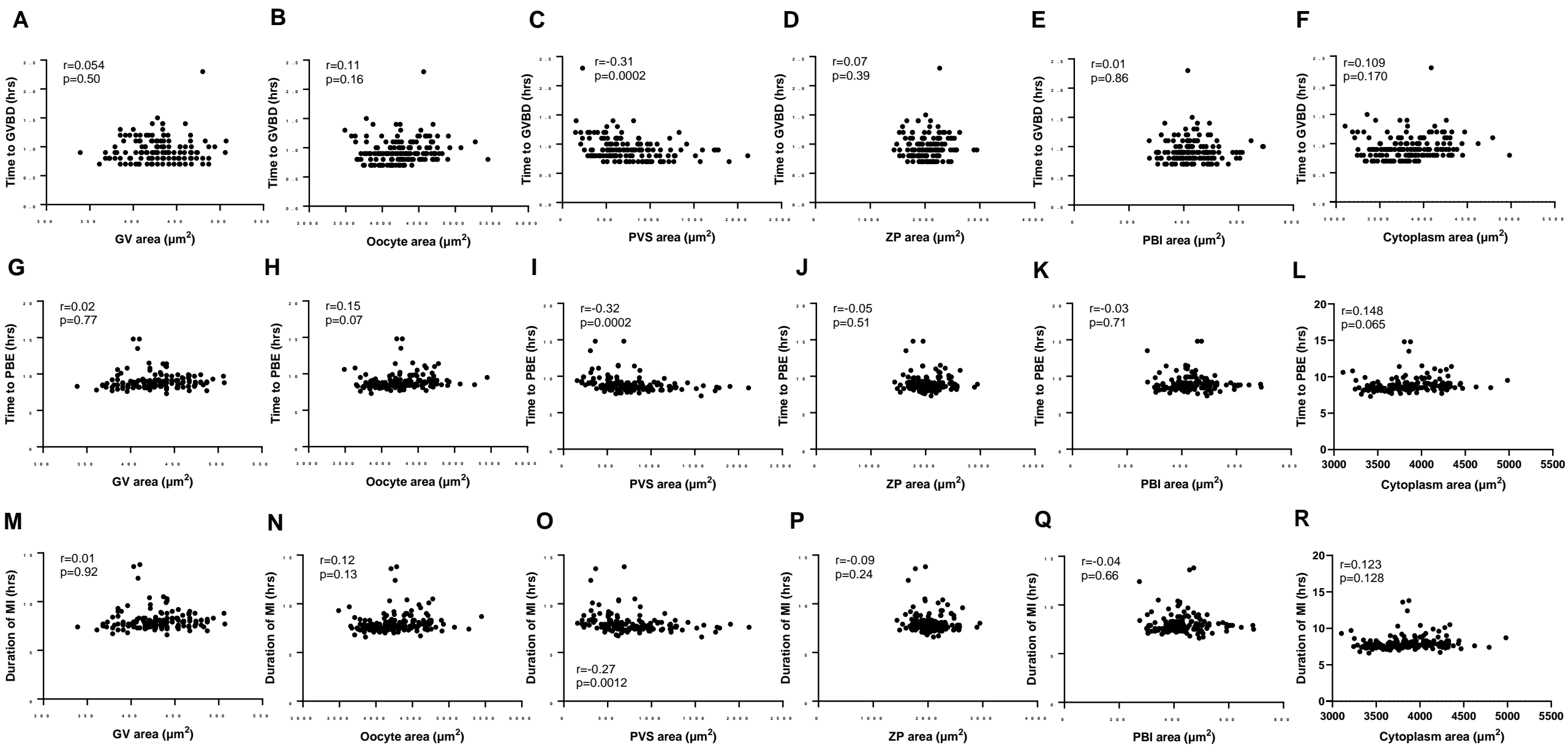

Supplement: Suebthawinkul-supplemantal_figures-3_ioac139 [file suebthawinkul-supplemantal_figures-3_ioac139.pdf]

Supplemental Figure 4

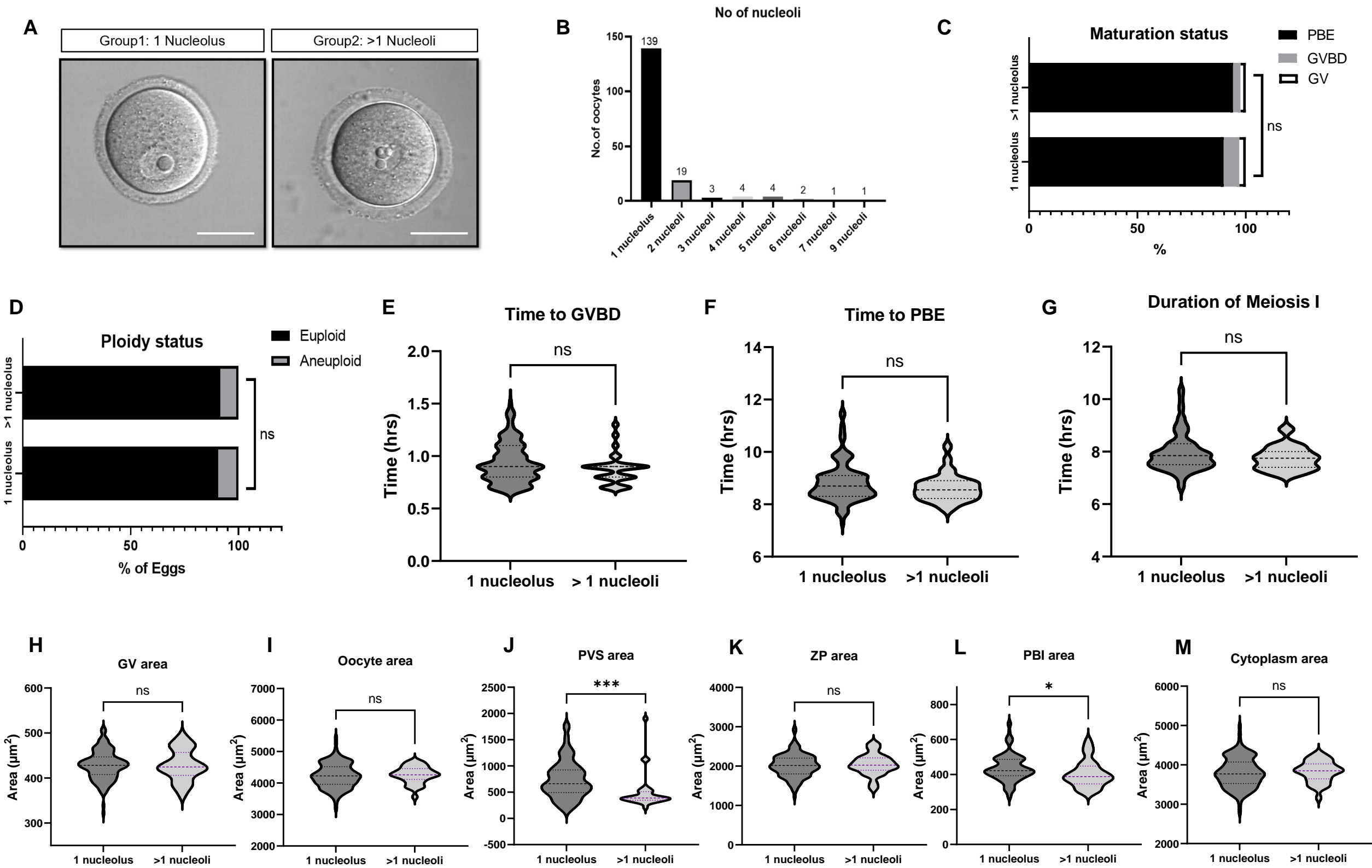

Supplement: Suebthawinkul-supplemantal_figures-4_ioac139 [file suebthawinkul-supplemantal_figures-4_ioac139.pdf]

# Supplemental Figure 5

50 nM Nocodazole

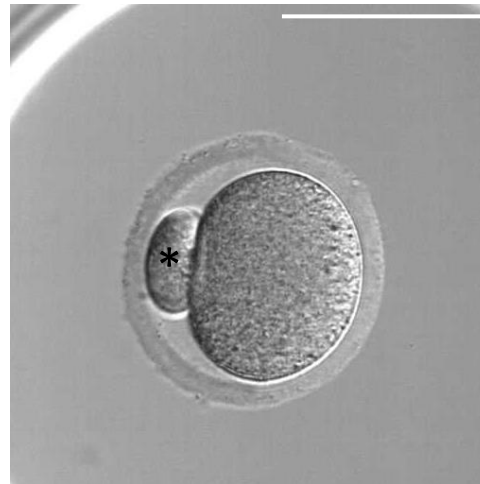

Normal (11.11%)

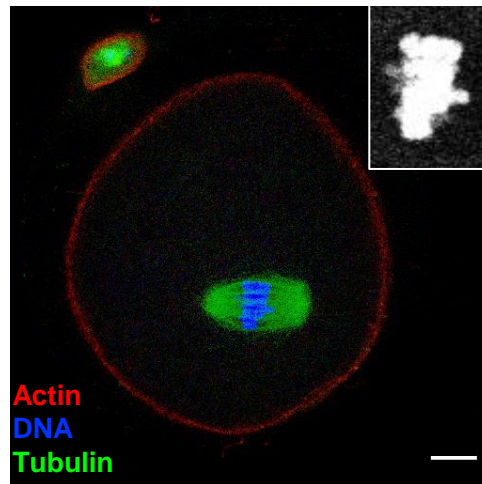

Abnormal (88.89%)

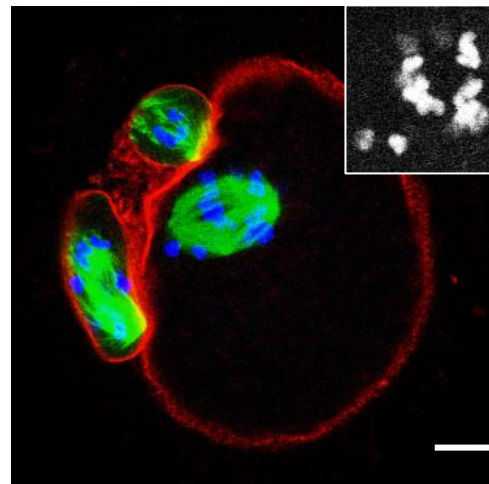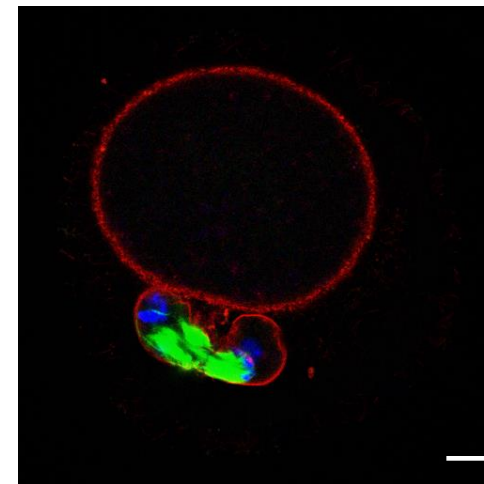

Supplement: Suebthawinkul-supplemantal_figures-5_ioac139 [file suebthawinkul-supplemantal_figures-5_ioac139.pdf]

Supplemental Figure 6

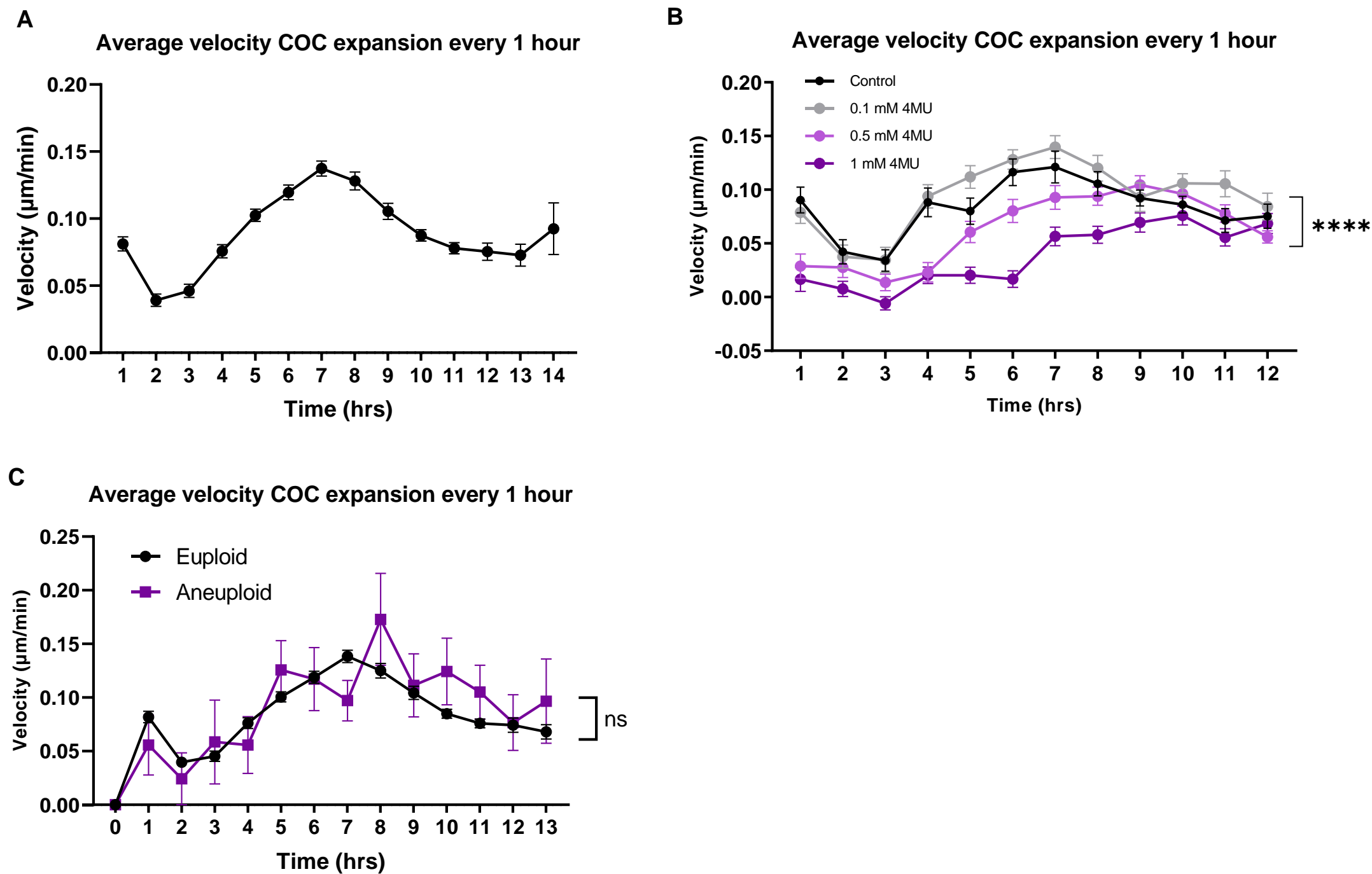

Supplement: Suebthawinkul-supplemantal_figures-6_ioac139 [file suebthawinkul-supplemantal_figures-6_ioac139.pdf]
