# Supplementary material for: Quantitative morphokinetic parameters identify novel dynamics of oocyte meiotic maturation and cumulus expansion
Source: Biol Reprod. 2022 Jul 8;107(4):1097–112. doi: 10.1093/biolre/ioac139 (PMC9562117; doi:10.1093/biolre/ioac139)
Supplement: Suebthawinkul_Supplemental_Table_ioac139 [file suebthawinkul_supplemental_table_ioac139.docx]

**Supplemental Table 1.** Maturation rate, euploidy rate, morphological and morphokinetic parameters of meiotic progression in 2 groups of oocytes based on the nucleolar number (Mean±SEM)

GVBD; germinal vesicle breakdown, PBE; polar body extrusion, GV; Germinal vesicle, PVS; perivitelline space, ZP; zona pellucida, PBI; first polar body, SEM; standard error of the mean

| **Parameters** | **1 nucleolus (n=138)** | **> 1 nucleoli (n=34)** | **P value** |
| --- | --- | --- | --- |
| PBE (%) | 89.78% | 94.12% | 0.742 |
| Euploidy Rate (%) | 90.48% | 91.67% | >0.9999 |
| Time to GVBD (hr) | 0.93 ± 0.02 | 0.87 ± 0.02 | 0.127 |
| Time to PBE (hr) | 8.833 ± 0.07 | 8.622 ± 0.09 | 0.151 |
| Duration of Meiosis I (hr) | 7.950 ± 0.07 | 7.750 ± 0.08 | 0.223 |
| GV size (µm^2^) | 428.0 ± 2.75 | 428.7 ± 5.92 | 0.909 |
| Oocyte size (µm^2^) | 4233.19 ± 33.35 | 4261.18 ± 45.88 | 0.693 |
| PVS area (µm^2^) | 736.75 ± 21.64 | 495.40 ± 60.86 | <0.0001 |
| ZP area (µm^2^) | 2013.76 ± 24.80 | 2038.74 ± 46.51 | 0.65 |
| PBI size (µm^2^) | 436.78 ± 6.93 | 402.61 ± 13.63 | 0.0085 |
| Cytoplasm area (µm^2^) | 3805.17 ± 32.46 | 3832.44 ± 45.88 | 0.694 |

**Supplemental Table 2.** Summary parameters of denuded oocytes and COCs from the validation experiments with nocodazole and 4MU (Mean±SEM)

| **Parameters of denuded oocytes** | **Nocodazole (n=190)** | | | | | | | | **P-value** |
| --- | --- | --- | --- | --- | --- | --- | --- | --- | --- |
|  | **Control** | **12.5 nM** | | **25 nM** | | **50 nM** | | **75 nM** |  |
| PBE rate (%) | 97.78 ± 2.22% | 93.54 ± 0.21% | | 96.88 ± 3.13% | | 18.75 ± 3.61% | | 0% | <0.0001 |
| Normal spindle and chromosome alignment (MII eggs) | 81.25% | 61.54% | | 53.85% | | 11.11% | |  | 0.0012 |
| Time to GVBD (hr) | 1.05 ± 0.03 | 1.01 ± 0.04 | | 0.95± 0.02 | | 1.02 ± 0.06 | | 1.10 ± 0.04 | 0.252 |
| Time to PBE (hr) | 8.85 ± 0.11 | 8.81 ± 0.11 | | 9.22 ± 0.17 | | 14.89 ± 0.26 | |  | <0.0001 |
| Duration of Meiosis I (hr) | 7.81 ± 0.09 | 7.60 ± 0.28 | | 8.27 ± 0.16 | | 14.08 ± 0.25 | |  | <0.0001 |
| **Parameters of COCs** | **4MU (n=172)** | | | | | | | | **P-value** |
|  | **Pre-IVM** | **Control** | | **0.1 mM** | | **0.5 mM** | | **1 mM** |  |
| Maturation rate |  | 97.92 ± 2.08% | | 97.92 ± 2.08% | | 69.30 ± 8.67% | | 2.22 ± 2.22% | <0.0001 |
| HA level  (Mean fluorescence intensity/pixel) | 59.33 ± 6.92 | 167.1 ± 2.88 | | 136.0 ± 1.85 | | 101.8 ± 2.76 | | 94.68 ± 2.50 | <0.0001 |
| Overall rate of cumulus expansion (µm/min) |  | 0.090 ± 0.003 | | 0.087 ± 0.003 | | 0.060 ± 0.003 | | 0.038 ± 0.002 | <0.0001 |
| **Parameters of denuded oocytes** | **4MU (n=172)** | | | | | | | | **P-value** |
|  | **Control** | | **0.1 mM** | | **0.5 mM** | | **1 mM** | |  |
| PBE rate (%) | 94.17 ± 0.84 | | 82.24 ± 7.24 | | 44.38 ± 0.63 | | 0% | | <0.0001 |
| Time to GVBD (hr) | 0.98 ± 0.03 | | 1.16 ± 0.06 | | 2.08 ± 0.33 | |  | | <0.0001 |
| Time to PBE (hr) | 9.11 ± 0.10 | | 9.61 ± 0.21 | | 11.42 ± 0.56 | |  | | <0.0001 |
| Duration of Meiosis I (hr) | 8.15 ± 0.10 | | 8.38 ± 0.13 | | 9.26 ± 0.42 | |  | | 0.0007 |
| Normal spindle and chromosome alignment (MII eggs) | 78.10 ± 1.91 | | 72.50 ± 2.50 | | 64.10 ± 2.56 | |  | | 0.365 |

GVBD; germinal vesicle breakdown, PBE; polar body extrusion, MII; metaphase of meiosis II, GV; Germinal vesicle, PVS; perivitelline space, ZP; zona pellucida, PBI; first polar body, COCs; cumulus-oocyte complexes, HA; hyaluronic acid, SEM; standard error of the mean
